# Supplementary material for: High thermoelectric efficiency realized in SnSe crystals via structural modulation
Source: Nat Commun. 2023 Mar 13;14:1366. doi: 10.1038/s41467-023-37114-7 (PMC10011372; doi:10.1038/s41467-023-37114-7)
Supplement: Supplementary file 1 — Supplementary Information [file 41467_2023_37114_MOESM1_ESM.pdf]

Supplementary Information

High thermoelectric efficiency realized in SnSe crystals via structural modulation

Bingchao Qin[1], Dongyang Wang[2], Tao Hong[1], Yuping Wang[1], Dongrui Liu[1], Ziyuan Wang[3], Xiang Gao[4], Zhen-Hua Ge[3], Li-Dong Zhao[1,*]

[1]School of Materials Science and Engineering, Beihang University, Beijing 100191, China

[2]Henan Key Laboratory of Diamond Optoelectronic Materials and Devices, Key Laboratory of Material Physics, Ministry of Education, School of Physics, Zhengzhou University, Zhengzhou 450052, China

[3]Faculty of Materials Science and Engineering, Kunming University of Science and Technology, Kunming 650093, China

[4]Center for High Pressure Science and Technology Advanced Research (HPSTAR), Beijing 100094, China

These authors contributed equally: Bingchao Qin, Dongyang Wang, Tao Hong.

[*]Corresponding author Email: zhaolidong@buaa.edu.cn

Calculations and Simulational Details

Density functional theory (DFT) calculations: The DFT calculations were performed using the projector augmented wave (PAW) method as implemented in Vienna Ab-initio Simulation Package (VASP) [1, 2]. The exchange-correlation energy is treated in the generalized gradient approximation (GGA) of Perdew-Burke-Ernzerhof (PBE).[2] The plane-wave cutoff energy sets to 500 eV. A 4 × 10 × 10 Monkhorst-Pack grids was used for the ***k***-point sampling. The temperature dependent electronic band structures of SnSe-9%Pb-1.2%Sr crystals were shown in **Supplementary Fig. 2**, calculated by using the experimental structures at elevated temperatures (from 300 K to 873 K) deriving from the refined SR-XRD data.

Theoretical simulations on the thermoelectric transport parameters from *Pnma* to *Cmcm* phases and multiband simulations.

Due to the non-parabolic feature of valence band, we adopted the Kane band model to evaluate the transport properties, including Seebeck coefficient and carrier mobility. In this work, the acoustic and polar optical phonon scattering were considered. Total relaxation time (τ) can be obtained by considering the acoustic phonon-electron scattering and the optical phonon scattering:

$$\tau^{-1} = \tau_{\mathrm{ac}}^{-1} + \tau_{op}^{-1} \tag{S1}$$

The relaxation time (τ_{ac}) of acoustic phonon-electron scattering based on deformation potential (DP) theory can be expressed as[3]:

$$\tau_{ac} = \frac{\pi\hbar^4 C_l N_v}{2^{1/2} {m_d^*}^{3/2} (k_B T)^{3/2} E_d^2} (\varepsilon + \beta\varepsilon^2)^{-1/2} (1 + 2\beta\varepsilon)^{-1} \left[1 - \frac{8\beta(\varepsilon+\beta\varepsilon^2)}{3(1+2\beta\varepsilon)^2}\right]^{-1} \tag{S2}$$

Here, k_B, $\hbar$, and ζ are the Boltzmann constant, reduced Plank constant, and reduced Fermi energy, respectively. $\beta = k_B T/E_g$, E_g is the energy bandgap of SnSe (E_g = 0.86 eV). C_l is the longitudinal elastic modulus (C_l = 74 GPa). E_d is deformation potential coefficient, defined as $\partial E/\partial(l/l_0)$, where E is the energy level of valley and l_0 is the equilibrium lattice constant. A series of lattice constants were used. In general, the strain on lattice will lead to a shift of reference energy level, which lead to a difficult in theoretical calculation of the absolute deformation potential. Wei and Zunger proposed the approach that assumes the energy level of the deep core state is not sensitive to the

slight lattice deformation[4]. The 1*s* core level of Sn was adopted as a reference energy to give absolute deformation potential. The calculated deformation potential for the four valleys is 9.8, 13.5, 7.9 and 13.0 eV, respectively.

The relaxation time (τ_{op}) of polar optical phonon-electron scattering can be expressed as[3]:

$$\tau_{op} = \frac{4\pi\hbar^2 N_v^{\frac{1}{3}}}{2^{\frac{1}{2}} m_d^{*\frac{1}{2}} e^2 (k_B T)^{\frac{1}{2}} (\varepsilon_\infty^{-1} - \varepsilon_s^{-1})} (1+2\beta\varepsilon)^{-1} (\varepsilon+\beta\varepsilon^2)^{\frac{1}{2}} \times A \quad \text{(S3)}$$

$$A = \left\{ \left[1 - \delta \, ln\left(1+\frac{1}{\delta}\right)\right] - \frac{2\beta(\varepsilon+\beta\varepsilon^2)}{(1+2\beta\varepsilon)^2} \left[1 - 2\delta + 2\delta^2 \, ln\left(1+\frac{1}{\delta}\right)\right] \right\}^{-1} \quad \text{(S4)}$$

where ε_s and ε_∞ are static and high frequency dielectric constant and set to 17ε_0 and 72ε_0, respectively, and ε_0 is the vacuum dielectric constant. δ is a function of reduced energy (ε), and it is defined as[3]:

$$\delta(\varepsilon) = \frac{e^2 m_d^{*\,1/2} N_v^{2/3}}{2^{1/2} \varepsilon (k_B T)^{1/2} \pi\hbar\varepsilon_\infty} (1+\beta\varepsilon)^{-1} \, {}^0F_1^{1/2}(\zeta,\beta) \quad \text{(S5)}$$

${}^lF_n^m$ (ζ, β)is generalized Fermi integral:

$${}^lF_n^m(\zeta,\beta) = \int_0^\infty \left(-\frac{\partial f}{\partial \varepsilon}\right) \varepsilon^l (\varepsilon+\beta\varepsilon^2)^m (1+2\beta\varepsilon)^n d\varepsilon \quad \text{(S6)}$$

$f(\zeta, \varepsilon)$the Fermi-Dirac distribution function:

$$f(\zeta,\varepsilon) = \frac{1}{e^{\left(\frac{\varepsilon-\zeta}{k_B T}\right)}+1} \quad \text{(S7)}$$

Based on above discussions, the total relaxation can be obtained. The carrier concentration, carrier mobility, Seebeck coefficient and Lorenz number of single Kane band model can be calculated[3]:

$$n = \frac{(2m_d^* k_B T)^{3/2}}{3\pi^2\hbar^3} \int_0^\infty \left(-\frac{\partial f}{\partial \varepsilon}\right) (\varepsilon+\beta\varepsilon^2)^{3/2} d\varepsilon \quad \text{(S8)}$$

$$\mu = \frac{e}{m_I^*} \frac{\int_0^\infty \left(-\frac{\partial f}{\partial \varepsilon}\right) \tau \frac{(\varepsilon+\beta\varepsilon^2)^{3/2}}{1+2\beta\varepsilon} d\varepsilon}{\int_0^\infty \left(-\frac{\partial f}{\partial \varepsilon}\right) (\varepsilon+\beta\varepsilon^2)^{3/2} d\varepsilon} \quad \text{(S9)}$$

$$S = \frac{k_B}{e} \left(\frac{\int_0^\infty \left(-\frac{\partial f}{\partial \varepsilon}\right) \tau \frac{\varepsilon(\varepsilon+\beta\varepsilon^2)^{3/2}}{1+2\beta\varepsilon} d\varepsilon}{\int_0^\infty \left(-\frac{\partial f}{\partial \varepsilon}\right) \tau \frac{(\varepsilon+\beta\varepsilon^2)^{3/2}}{1+2\beta\varepsilon} d\varepsilon} - \zeta \right) \quad \text{(S10)}$$

$$L = \left(\frac{k_B}{e}\right)^2 \left\{ \frac{\int_0^\infty \left(-\frac{\partial f}{\partial \varepsilon}\right) \tau \frac{\varepsilon^2(\varepsilon+\beta\varepsilon^2)^{3/2}}{1+2\beta\varepsilon} d\varepsilon}{\int_0^\infty \left(-\frac{\partial f}{\partial \varepsilon}\right) \tau \frac{(\varepsilon+\beta\varepsilon^2)^{3/2}}{1+2\beta\varepsilon} d\varepsilon} - \left[\frac{\int_0^\infty \left(-\frac{\partial f}{\partial \varepsilon}\right) \tau \frac{\varepsilon(\varepsilon+\beta\varepsilon^2)^{3/2}}{1+2\beta\varepsilon} d\varepsilon}{\int_0^\infty \left(-\frac{\partial f}{\partial \varepsilon}\right) \tau \frac{(\varepsilon+\beta\varepsilon^2)^{3/2}}{1+2\beta\varepsilon} d\varepsilon} \right]^2 \right\} \quad \text{(S11)}$$

where m_b^* is the single valley effective mass ($m_b^* = (m_x^* m_y^* m_z^*)^{1/3}$, m_d^* is density of states (DOS) effective mass ($m_d^* = N_v^{3/2} m_b^*$), N_v is degeneracy of valence band (N_v = 2

for each band valley), and m_I^* is conductance effective mass (m_I^* = $3/(1/m_x^*+1/m_y^*+1/m_z^*)$).

For multiple valleys participated in transport, total carrier concentration (n_{tot}), the total carrier mobility (μ_{tot}), total Seebeck coefficient (S_{tot}), and total Lorenz number (L_{tot}) can be expressed as:

$$n_{tot} = \sum_i n_i \quad \text{(S12)}$$

$$\mu_{tot} = \frac{\sum_i n_i \mu_i}{\sum_i n_i} \quad \text{(S13)}$$

$$S_{tot} = \frac{\sum_i n_i \mu_i S_i}{\sum_i n_i \mu_i} \quad \text{(S14)}$$

$$L_{tot} = \frac{\sum_i n_i \mu_i L_i}{\sum_i n_i \mu_i} \quad \text{(S15)}$$

where n_i, μ_i, S_i, and L_i are the carrier concentration, carrier mobility, Seebeck coefficient, and Lorenz number contributed by the i-th valley. The relationship between VBM 1 and i-th valley can be obtained by $\zeta_i=\zeta-\Delta E_i$ and $\beta_i=k_BT/(E_g+\Delta E_i)$, ΔE_i is the energy difference between VBM1 and i-th valley. By using the electronic thermal conductivity evaluated through Wiedemann-Franz ($\kappa_{ele}=L\sigma T$) and measured lattice thermal conductivity (κ_{lat}), we can calculate the carrier-dependent ZT.

In Seebeck coefficient, carrier mobility and ZT calculations, non-parabolic Kane multi-valley model is used. All the parameters, including DOS and conductance effective mass, energy difference, deformation potential (DP) and band degeneracy, are derived from DFT calculations based on the *Pnma* and *Cmcm* structures, respectively.

After we obtained the two end curves in **Fig. 1** by using the corresponding electronic band structures of full-*Pnma* and full-*Cmcm* phases from SR-XRD results. For the intermediate states between *Pnma* and *Cmcm* phases, we also calculated the performance parameters by using their corresponding electronic band structures. To establish the connection between the phase compositions and band structures, we consider the chosen angle 1 shown in **Fig. 4**. First of all, the angles were determined to be ~ 7.8° and 0 for full-*Pnma* phase and full-*Cmcm* phase, respectively. Secondly, we chose an intermediate temperature, obtained its angle in the crystal structure, and thus determined the phase composition between full-*Pnma* phase and full-*Cmcm* phase with its corresponding band structure. For example, at 473 K, the angle was ~ 7.3° due to

the refined crystal structure, corresponding to the intermediate state with ~ 93.6% *Pnma* or 6.4% *Cmcm*. Therefore, the electronic band structure for SnSe at 473 K was used to calculated the performance parameters of the intermediate state with 93.6% *Pnma* (also corresponding to 6.4% *Cmcm*). On this basis, several intermediate states between full-*Pnma* phase and full-*Cmcm* phase and their corresponding performance parameters were calculated, and we finally obtained the simulated 3D curves shown in **Fig. 1**.

Calculation for the heat capacity (C_p) based on Debye model.

Based on the Debye model, we considered the individual contributions of phonons and the effects of thermal expansion, to the total heat capacity of SnSe system[5, 6]. The total heat capacity, $C_{p,tot}$, as a function of temperature, can be written as:

$$C_{p,tot}(T) = C_{p,ph}(T) + C_{p,D}(T) \tag{S16}$$

where $C_{p,ph}$ and $C_{p,D}$ represent the phonon heat capacity capacity and the effects of lattice dilation on the heat capacity capacity, respectively.

Here, due to the Debye assumption and elastic wave approximation, the phonon heat capacity capacity $C_{p,ph}$ can be obtained as:

$$C_{p,ph}(T/\Theta_D) = 9R(\frac{T}{\Theta_D})^3 \int_0^{\Theta_D/T} \frac{x^4 e^x}{(e^x-1)^2} dx \tag{S17}$$

where Θ_D refers to the Debye model, R = 8.314 Jmol^{-1}K^{-1}, and $x = \hbar\omega/k_BT$, in which $\hbar$, ω and k_B represent Planck constant, phonon vibration frequency and Boltzmann constant, respectively.

The effects of thermal expansion on the heat capacity capacity can be derived from the thermal expansion in a given system. The effects of lattice dilation on the total heat capacity capacity, $C_{p,D}$, can be obtained from:

$$C_{p,D}(T) = C_{ele,D}(T) + C_{ph,D}(T) = \frac{9BT\alpha^2}{10^6\rho} \tag{S18}$$

where B is the isothermal bulk modulus, α is the linear coefficient of thermal expansion, and ρ is the sample density (all dependent on temperature) (*67*). Based on the above discussion, we obtained total heat capacity for all samples from Debye model. It should be noted that the electrons also contribute to the heat capacity, as $C_{p,ele}$. However, since the effect of dilation is mostly phononic in origin, as $C_{p,ph}$ is much larger than $C_{p,ele}$, therefore, the contribution from electrons was not taken into account.

Calculations of average *ZT* (ZT_{ave}).

Among a given temperature range (300 - 773 K), the average *ZT* value (ZT_{ave}) is given by:

$$ZT_{\mathrm{ave}} = \frac{1}{T_{\mathrm{h}} - T_{\mathrm{c}}} \int_{T_{\mathrm{c}}}^{T_{\mathrm{h}}} ZT dT \quad \text{(S19)}$$

where T_h and T_c represent the hot and cold side temperature, respectively. In this work, the T_c is 300K, and T_h is 773K.

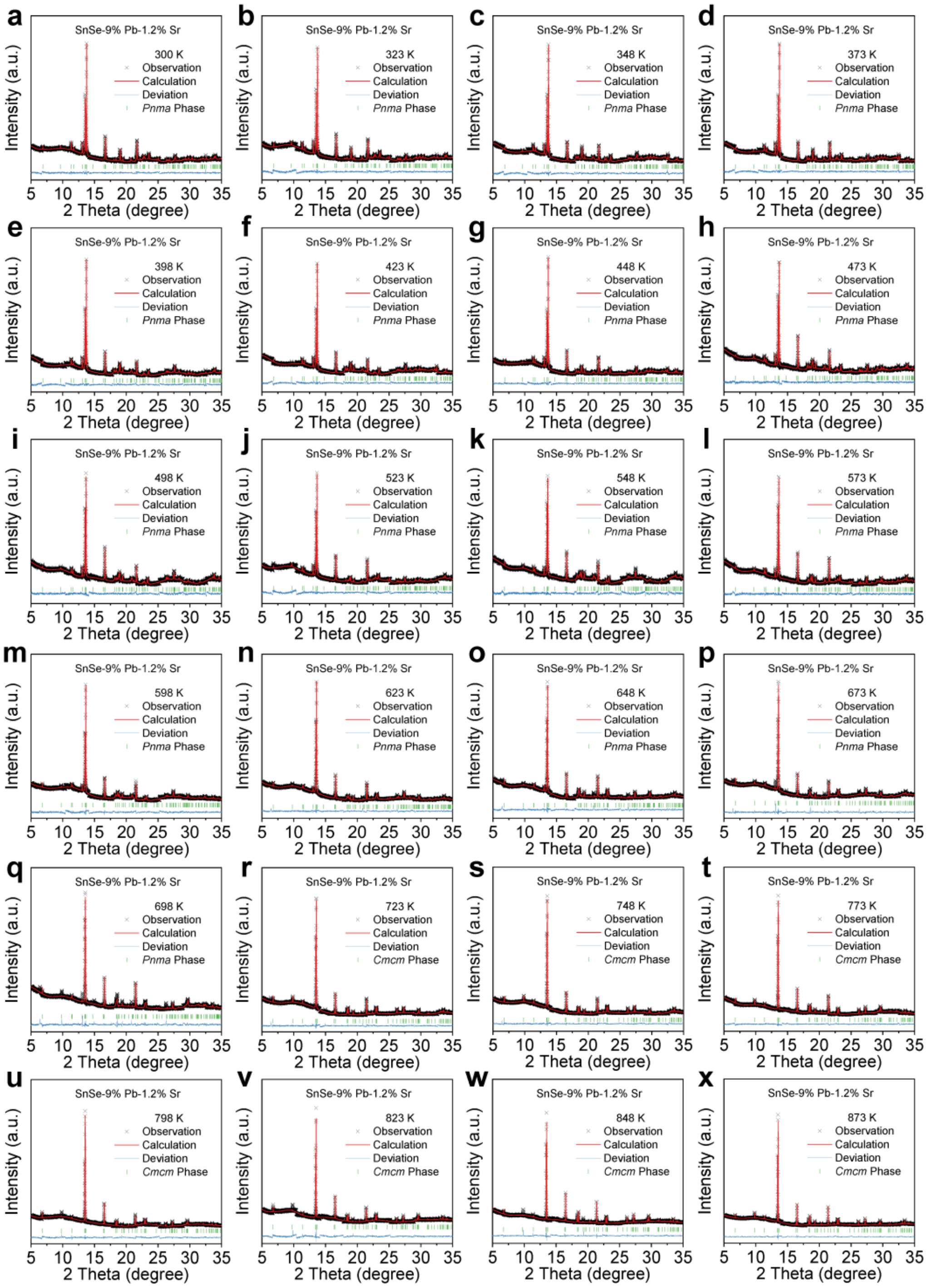

Supplementary Fig. 1. Temperature-dependent SR-XRD patterns and Rietveld refinements for p-type SnSe-9%Pb-1.2%Sr crystal from 300 K to 873 K. (a) 300 K; (b) 323 K; (c) 348 K; (d) 373 K; (e) 398 K; (f) 423 K; (g) 448 K; (h) 473 K; (i) 498 K; (j) 523 K; (k) 548 K; (l) 573 K; (m) 598 K; (n) 623 K; (o) 648 K; (p) 673 K; (q) 698 K; (r) 723 K; (s) 748 K; (t) 773 K; (u) 798 K; (v) 823 K; (w) 848 K; (x) 873 K.

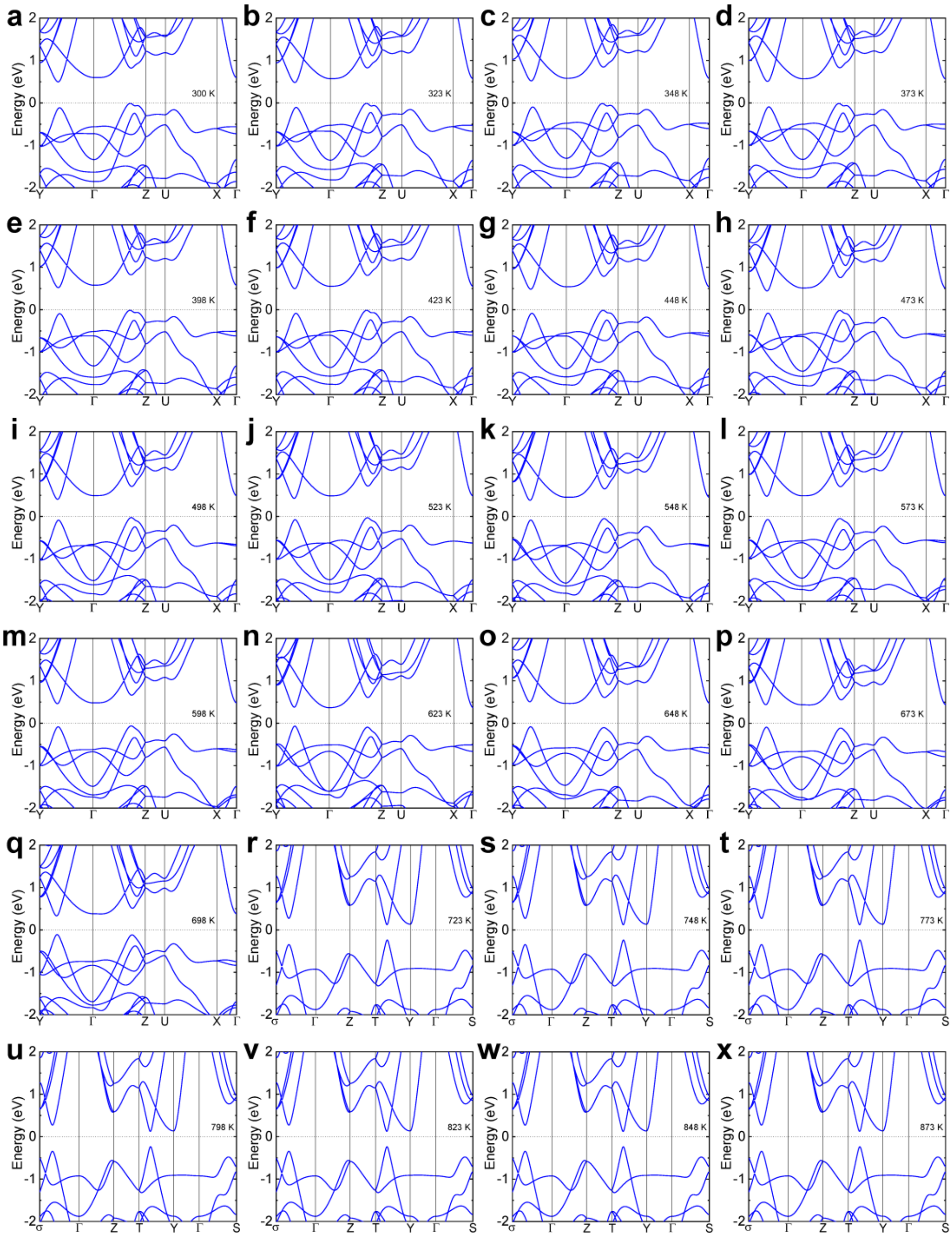

Supplementary Fig. 2. The calculated electronic band structures with rising temperature for p-type SnSe-9%Pb-1.2%Sr crystal from 300 K to 873 K. (a) 300 K; (b) 323 K; (c) 348 K; (d) 373 K; (e) 398 K; (f) 423 K; (g) 448 K; (h) 473 K; (i) 498 K; (j) 523 K; (k) 548 K; (l) 573 K; (m) 598 K; (n) 623 K; (o) 648 K; (p) 673 K; (q) 698 K; (r) 723 K; (s) 748 K; (t) 773 K; (u) 798 K; (v) 823 K; (w) 848 K; (x) 873 K.

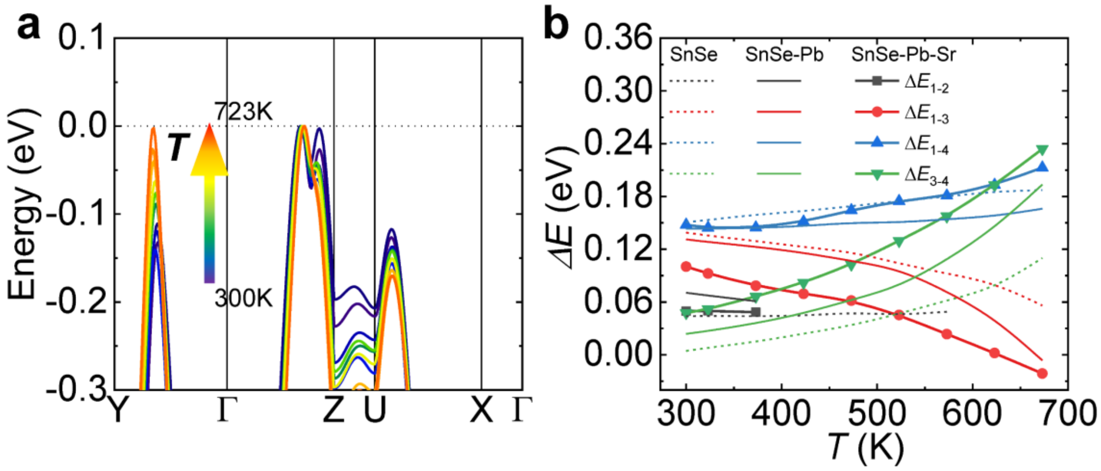

Supplementary Fig. 3. The multiple valence bands evolution with temperature for p-type SnSe. (a) The dynamic evolution of four separate valence band valleys with rising temperature for p-type SnSe-9%Pb-1.2%Sr crystal from 300 K to 723 K, where VBM 1 was aligned at each temperature, indicating that the VBM 3 along Γ-Y direction has arisen higher than VBM (1+2) along Γ-Z direction earlier than 723 K. (b) The energy differences (ΔE) between VBM 1 and VBM 2, VBM 1 and VBM 3, VBM 1 and VBM 4, and VBM 3 and VBM 4 as a function of temperature for p-type SnSe, SnSe-9%Pb, and SnSe-9%Pb-1.2%Sr crystals.

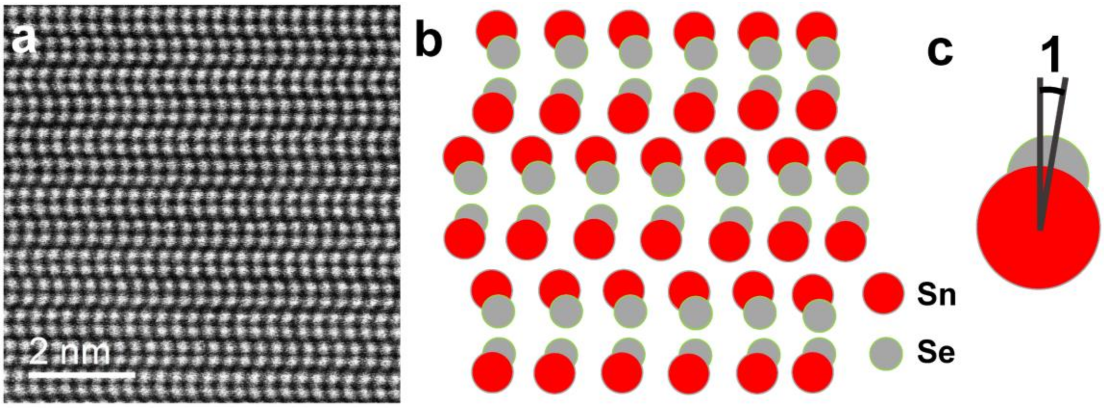

Supplementary Fig. 4. The *Pnma*-phase structure of SnSe. (a) The HAADF-STEM image for the undoped area with the *Pnma*-phase structure along the [110] axis; (b) The schematic diagram of the arrangement for Sn atoms and Se atoms along the [110] direction; (c) The angle between the Sn atom and Se atom marked with the black label.

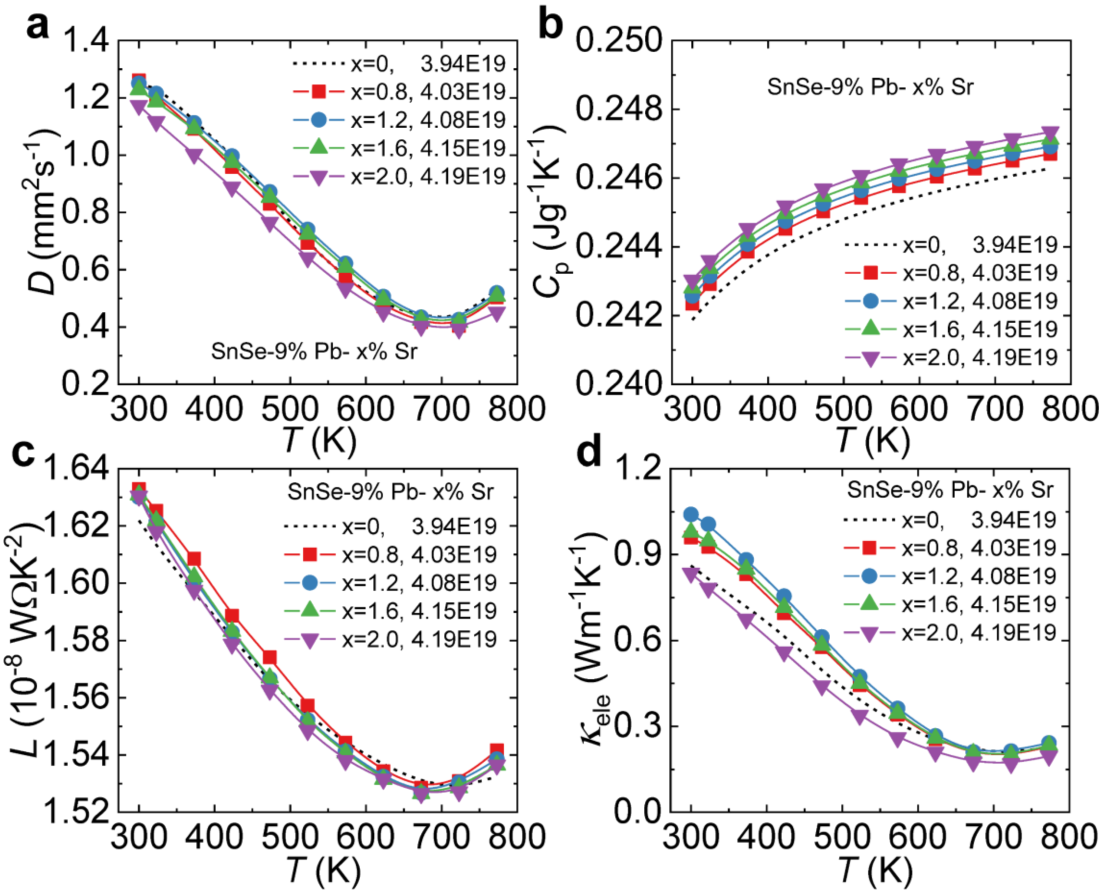

Supplementary Fig. 5. The thermoelectric transport properties as a function of temperature for SnSe-9%Pb-x%Sr crystals. (a) Thermal diffusivity (D). (b) Heat capacity (C_p). (c) Lorenz number (L). (d) Electronic thermal conductivity (κ_{ele}).

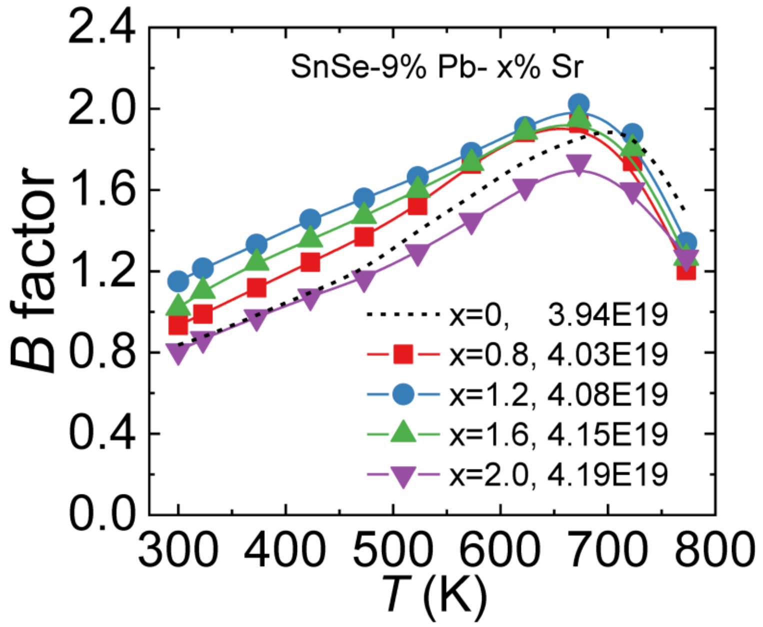

Supplementary Fig. 6. The quality factor *B* as a function of temperature for SnSe-9%Pb-x%Sr crystals.

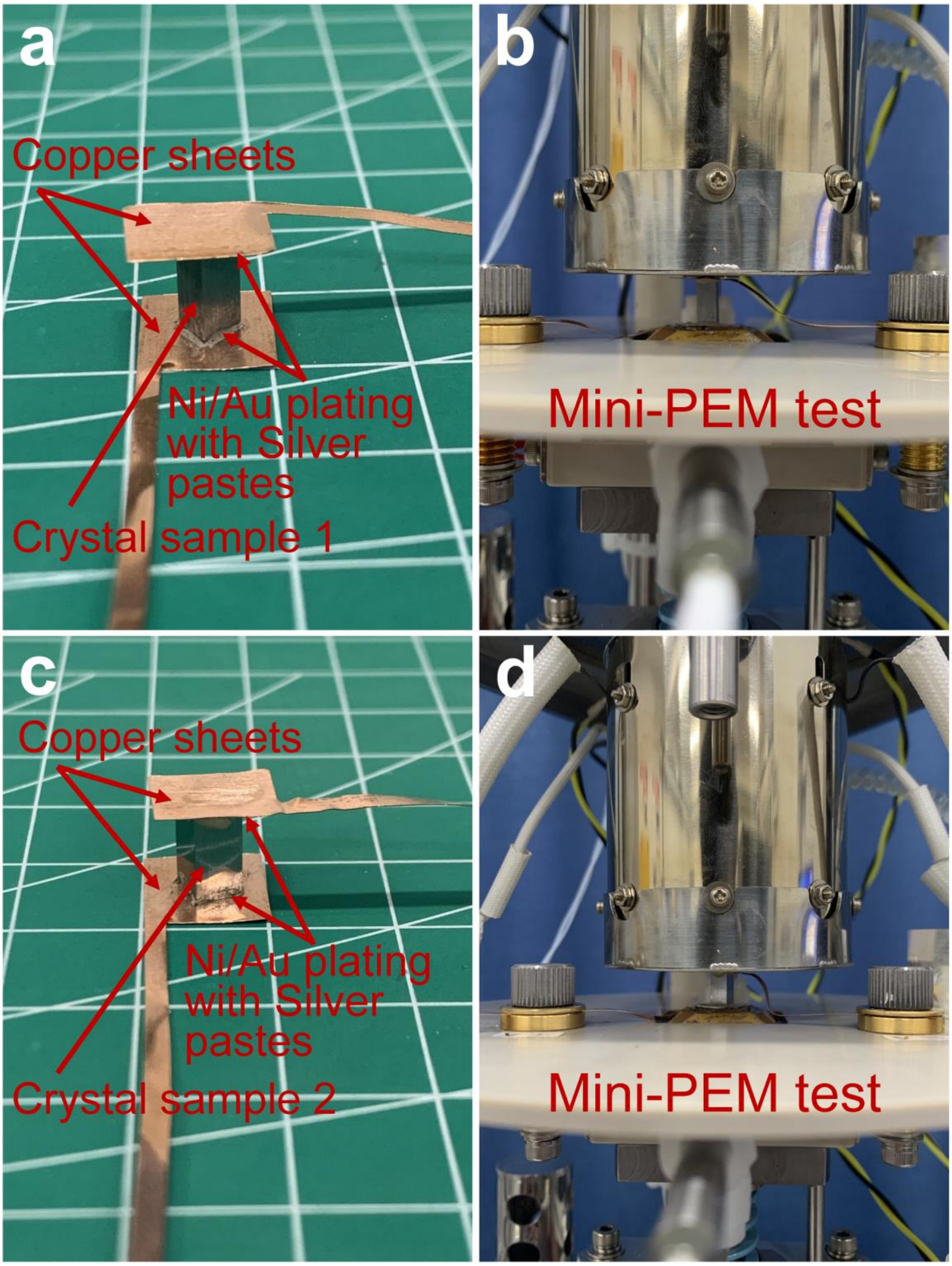

Supplementary Fig. 7. Fabrication and power generation performance measurement of the single-leg devices. (a) As-fabricated single-leg device 1; (b) Power generation performance measurement for single-leg device 1 by using Mini-PEM test system; (c) As-fabricated single-leg device 2; (d) Power generation performance measurement for single-leg device 2 by using Mini-PEM test system.

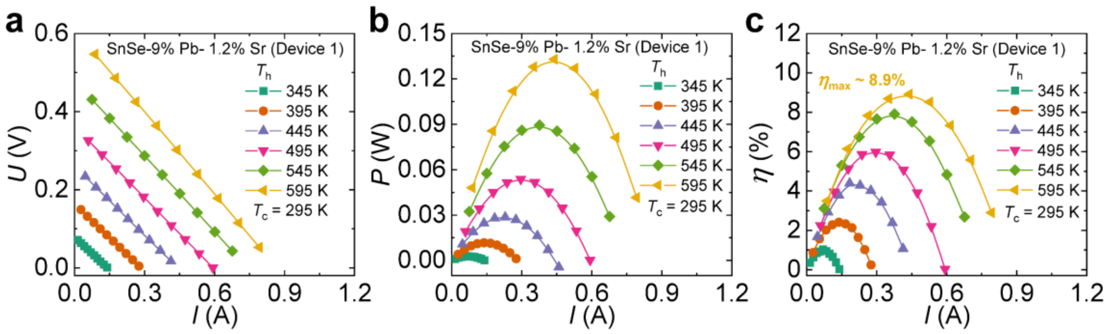

Supplementary Fig. 8. Power generation performance of the single-leg thermoelectric device (device 1) using SnSe-9%Pb-1.2%Sr crystals. (a) Voltage U, (b) Output power P, and (c) conversion efficiency η as a function of external current I at various temperature differences from 50 K to 300 K with the cold-end temperature T_c fixed at ~ 295 K.

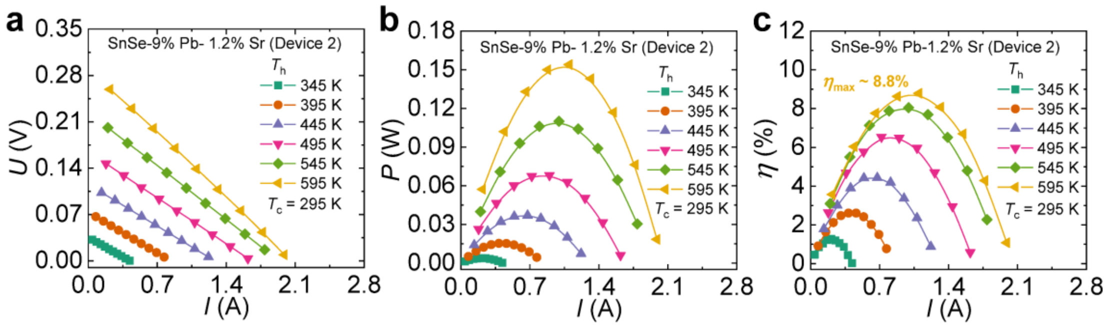

Supplementary Fig. 9. Power generation performance of the single-leg thermoelectric device (device 2) using SnSe-9%Pb-1.2%Sr crystals. (a) Voltage U, (b) Output power P, and (c) conversion efficiency η as a function of external current I at various temperature differences from 50 K to 300 K with the cold-end temperature T_c fixed at ~ 295 K.

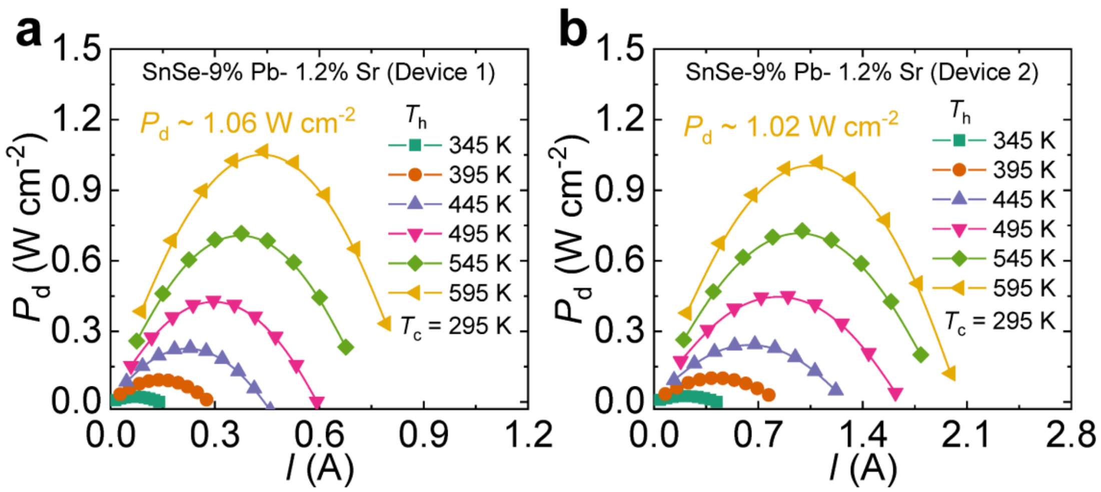

Supplementary Fig. 10. The output power density (P_d) as a function of external current I at various temperature differences from 50 K to 300 K with the cold-end temperature T_c fixed at ~ 295 K. (a) Device 1, and (b) Device 2.

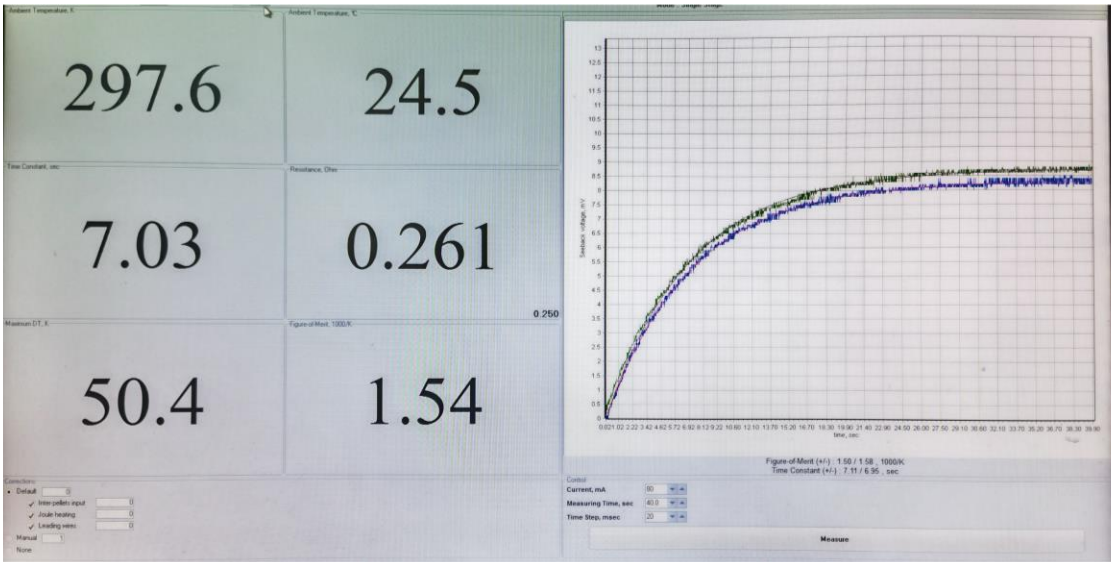

Supplementary Fig. 11. The thermoelectric cooling performance of the as-fabricated 7-pair thermoelectric device.

Supplementary Table 1. The refinement results consisting of lattice parameters for different crystal directions and atomic positions of Sn (Pb/Sr) and Se atoms in p-type SnSe-9%Pb-1.2%Sr crystals with increasing temperature from 300 K to 698 K.

T (K)	*a* (Å)	*b* (Å)	*c* (Å)	Sn-x	Sn-y	Sn-z	Se-x	Se-y	Se-z	χ^2
300	11.56695	4.19074	4.41512	0.12095	0.25	0.0915	0.85642	0.25	0.4824	0.9679
323	11.57438	4.19427	4.41414	0.11994	0.25	0.0895	0.85541	0.25	0.4803	1.164
348	11.58304	4.19906	4.41035	0.11876	0.25	0.0913	0.85423	0.25	0.4822	1.248
373	11.59048	4.20413	4.40581	0.11918	0.25	0.0901	0.85466	0.25	0.4810	1.095
398	11.60076	4.20873	4.40302	0.11947	0.25	0.0910	0.85494	0.25	0.4818	1.078
423	11.60913	4.21493	4.39700	0.11938	0.25	0.0885	0.85485	0.25	0.4793	1.119
448	11.61766	4.22051	4.39151	0.11988	0.25	0.0874	0.85535	0.25	0.4782	0.8629
473	11.62923	4.22771	4.38703	0.12030	0.25	0.0840	0.85577	0.25	0.4749	0.8461
498	11.63953	4.23555	4.38023	0.12142	0.25	0.0816	0.85689	0.25	0.4725	1.069
523	11.64814	4.24311	4.37380	0.12049	0.25	0.0821	0.85596	0.25	0.4730	1.254
548	11.66947	4.25759	4.36291	0.12156	0.25	0.0791	0.85703	0.25	0.4700	1.177
573	11.67064	4.26190	4.35544	0.11982	0.25	0.08524	0.85529	0.25	0.47608	0.8414
598	11.69086	4.27522	4.34913	0.12065	0.25	0.0846	0.85612	0.25	0.4755	1.170
623	11.69887	4.28983	4.32979	0.11868	0.25	0.0762	0.85415	0.25	0.4670	1.188
648	11.71115	4.30509	4.31577	0.12016	0.25	0.0877	0.85563	0.25	0.4785	1.368
673	11.72564	4.30992	4.31300	0.12156	0.25	0.0833	0.85703	0.25	0.4742	1.481
698	11.73692	4.29841	4.32823	0.12254	0.25	0.0764	0.85801	0.25	0.4673	1.805

Supplementary Table 2. The refinement results consisting of lattice parameters for different crystal directions and atomic positions of Sn (Pb/Sr) and Se atoms in p-type SnSe-9%Pb-1.2%Sr crystals with increasing temperature from 723 K to 873 K.

T (K)	a (Å)	b (Å)	c (Å)	Sn-x	Sn-y	Sn-z	Se-x	Se-y	Se-z	χ^2
723	4.29825	11.74163	4.33117	0.0	0.12239	0.25	0.5	0.85469	0.25	1.211
748	4.30084	11.74815	4.33309	0.0	0.12305	0.25	0.5	0.85535	0.25	1.098
773	4.30198	11.75409	4.33539	0.0	0.12308	0.25	0.5	0.85537	0.25	1.073
798	4.33918	11.76094	4.30370	0.0	0.12307	0.25	0.5	0.85537	0.25	1.337
823	4.34355	11.76869	4.30512	0.0	0.12563	0.25	0.5	0.85793	0.25	1.481
848	4.34861	11.77836	4.30672	0.0	0.12383	0.25	0.5	0.85613	0.25	1.431
873	4.30674	11.77875	4.34927	0.0	0.12246	0.25	0.5	0.85476	0.25	1.253

Supplementary Table 3. Sample densities for SnSe-9%Pb-x%Sr crystals.

Sample (x)	Density (g cm^{-3})
0	6.01
0.8	5.95
1.2	5.98
1.6	6.05
2.0	5.97

Supplementary References

1. Blöchl PE. Projector augmented-wave method. *Physical Review B* **50**, 17953-17979 (1994).
2. Kresse G, Furthmüller J. Efficient iterative schemes for ab initio total-energy calculations using a plane-wave basis set. *Physical Review B* **54**, 11169-11186 (1996).
3. Ravich YI, Efimova BA, Smirnov IA. *Semiconducting lead chalcogenides*. Springer Science & Business Media (1970).
4. Wei S-H, Zunger A. Predicted band-gap pressure coefficients of all diamond and zinc-blende semiconductors: Chemical trends. *Physical Review B* **60**, 5404-5411 (1999).
5. Debye P. Zur theorie der spezifischen wärmen. *Annalen der Physik* **344**, 789-839 (1912).
6. Qin B*, et al.* Realizing High Thermoelectric Performance in p-Type SnSe through Crystal Structure Modification. *Journal of the American Chemical Society* **141**, 1141-1149 (2019).
